# The binding selectivity of the C-terminal SH3 domain of Grb2, but not its folding pathway, is dictated by its contiguous SH2 domain

**DOI:** 10.1016/j.jbc.2024.107129

**Published:** 2024-03-01

**Authors:** Mariana Di Felice, Livia Pagano, Valeria Pennacchietti, Awa Diop, Paola Pietrangeli, Lucia Marcocci, Sara Di Matteo, Francesca Malagrinò, Angelo Toto, Stefano Gianni

**Affiliations:** 1 Dipartimento di Scienze Biochimiche “A. Rossi Fanelli”, Sapienza Università di Roma, Laboratory Affiliated to Istituto Pasteur Italia - Fondazione Cenci Bolognetti, Rome, Italy; 2 Dipartimento di Medicina clinica, sanità pubblica, scienze della vita e dell'ambiente, Università dell’Aquila, L'Aquila, Coppito, Italy

**Keywords:** protein–protein interaction, kinetics, mutagenesis, double-mutant cycle, multidomain protein

## Abstract

The adaptor protein Grb2, or growth factor receptor–bound protein 2, possesses a pivotal role in the transmission of fundamental molecular signals in the cell. Despite lacking enzymatic activity, Grb2 functions as a dynamic assembly platform, orchestrating intracellular signals through its modular structure. This study delves into the energetic communication of Grb2 domains, focusing on the folding and binding properties of the C-SH3 domain linked to its neighboring SH2 domain. Surprisingly, while the folding and stability of C-SH3 remain robust and unaffected by SH2 presence, significant differences emerge in the binding properties when considered within the tandem context compared with isolated C-SH3. Through a double mutant cycle analysis, we highlighted a subset of residues, located at the interface with the SH2 domain and far from the binding site, finely regulating the binding of a peptide mimicking a physiological ligand of the C-SH3 domain. Our results have mechanistic implications about the mechanisms of specificity of the C-SH3 domain, indicating that the presence of the SH2 domain optimizes binding to its physiological target, and emphasizing the general importance of considering supramodular multidomain protein structures to understand the functional intricacies of protein–protein interaction domains.

Grb2, also known as growth factor receptor–bound protein 2, is a widely distributed and highly conserved adaptor protein. Its primary role is to relay signals from activated receptor tyrosine kinases (RTKs) to downstream effectors. Grb2 was initially discovered through its interaction with tyrosine-phosphorylated RTKs, such as the epidermal growth factor receptor and the platelet-derived growth factor receptor (1, 2). Subsequently, it was found to be a crucial component of the mitogen-activated protein kinase signaling cascade. When epidermal growth factor receptor is activated, Grb2 binds to the guanine nucleotide exchange factor SOS and relocates SOS to the plasma membrane. This action triggers SOS-mediated activation of membrane-anchored Ras (3, 4). Over time, Grb2 has been revealed to interact with numerous other proteins. These include various RTKs as well as transmembrane and cytosolic adaptor proteins, GEFs, GTPases, and E3 ubiquitin ligases. Notably, Grb2 is involved in signaling processes that not only promote cell growth and differentiation but also play a role in actin cytoskeletal rearrangement and endocytosis (5, 6).

Despite its critical role in several key steps of cell metabolism, Grb2 lacks any enzymatic activity and exerts its functions by acting as a dynamic assembly platform that integrates various intracellular signals transduced by diverse cell surface receptors (1). This function is achieved; thanks to its modular nature that comprises three contiguous protein–protein recognition domains consisting of an SH2 domain flanked by two SH3 domains at its N- and C-terminal ends, namely N-SH3 and C-SH3. By recognizing specific partners, these recognition domains allow Grb2 to recruit large multiprotein complexes, thereby consolidating diverse intracellular signals (5, 7, 8).

A problem of interest pertaining docking proteins composed of different protein–protein recognition modules lies in understanding the energetic communication, if any, of its constituent domains. In fact, whilst it may be shallowly assumed that each individual domain functions in isolation in a similar manner as it may be observed in more complex constructs, there are indications that the presence of a contiguous domain may fine-tune the affinity for specific ligands (9). These effects may also influence the folding and stability of a protein domain, which may differ in isolation as compared with what may be measured in the context of the whole protein system (10). In particular, in the case of Grb2, it has been proposed that the interface between the SH2 domain and the C-terminal SH3 domain may play a role in the binding events mediated by the latter that may work as a supertertiary module (11). These hypotheses encourage engaging a detailed experimental examination.

We have recently characterized in detail both the folding and binding properties of the C-SH3 from Grb2 by employing kinetic experiments in synergy with extensive site-directed mutagenesis (12, 13, 14). The C-SH3 is particularly important as it mediates the binding between Grb2 with Gab1 and Gab2, an interaction that appears to be upregulated in several forms of human cancers (5, 15, 16, 17). In this work, we resorted to investigate the robustness of the folding and binding properties of C-SH3 by comparing the previously existing data with experiments carried out in the presence of the flanking SH2 domain. Surprisingly, we find that, despite the folding and stability of C-SH3 are highly robust and essentially unaffected by the presence of SH2, there are profound differences in the binding properties of the domain when considered in a more complex construct. Furthermore, at variance with what previously measured on the isolated domain, we demonstrate that, in analogy to what previously observed on PDZ domain (18), the whole protein moiety of the C-SH3 domain appears to be optimized to bind its physiological target. This behavior has been previously invoked to explain the observed specificity of protein–protein interaction domains.

## Results

An interesting question arising from recent literature is whether the molecular mechanisms of folding and binding of protein domains is robust and conserved when they are considered in isolation, as compared with their behavior in more complex architectures (10, 19, 20, 21, 22). As briefly recapitulated previously, the case of Grb2 is particularly instructive as this protein represents a critical target for several types of human cancers, and the folding and function of its C-terminal SH3 domain has been extensively characterized.

To test the robustness of the folding and functional behavior of C-SH3, we resorted to compare previously existing data, with a detailed mutational study on a more complex construct comprising the SH2 domain and C-SH3. For the sake of clarity, we will first focus on the folding data and subsequently describe the binding properties of the SH2–SH3 tandem.

### The folding of C-SH3 is robust and conserved in the SH2–SH3 tandem

The folding pathway of C-SH3 is a highly co-operative reaction that conforms to a simple two-state scenario (13). Hence, to investigate the robustness of C-SH3 folding, we conducted an analysis based on Φ values (23). This approach is conceived to infer residue-specific structural insights of intermediates and transition states along the folding pathway. To achieve this, we compared the folding kinetics of the WT protein with a series of conservative single mutants. Quantitatively, the Φ value is obtained for a given mutant by normalizing the stability change of the transition state, relative to that of the native state. A Φ value approximating 1 suggests a native-like structure in the transition state, whereas a Φ value of 0 indicated that the mutated residue resembled the denatured state in terms of structure.

We generated 18 site-specific variants of C-SH3, which were subsequently produced, expressed, and subjected to folding and unfolding experiments. These mutants were designed in accordance with the established principles of Φ value analysis, extensively discussed elsewhere. In essence, our design involved a conservative deletion of hydrophobic side chains, a mutation type that offers clear interpretability.

**Figure 1:**
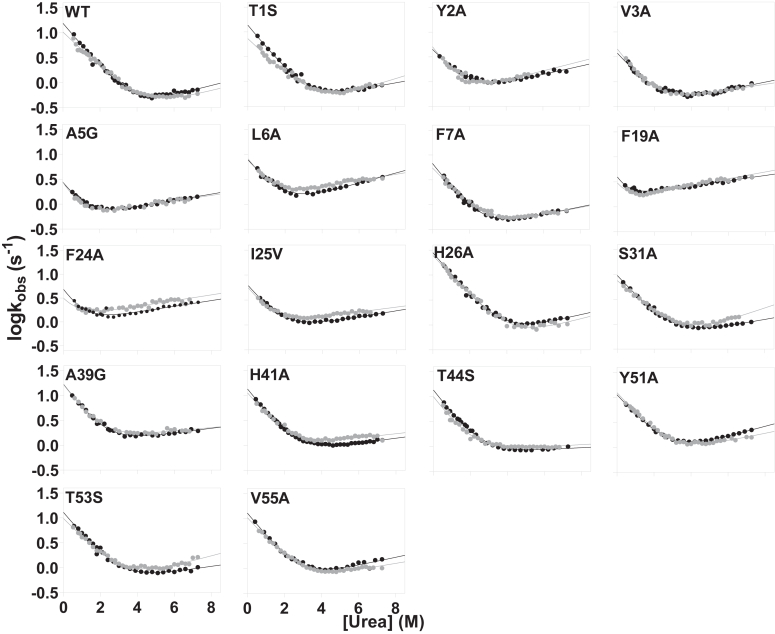
**Chevron plots of site directed mutants of the SH2–SH3 tandem (in *gray*) compared with mutants of isolated C-SH3 domain (in *black*) (data taken from Ref.** (12)**).***Lines* represent the best fit to Equation 1.

The folding and unfolding kinetics of SH2–SH3 was investigated by stopped-flow experiments. In analogy to our previous work, experiments were performed at 25 °C in 50 mM sodium phosphate buffer at pH 7.2 (13). We recently successfully assigned the kinetic phases associated to the individual domains of Grb2 in comparison to the tandem protein, which allowed us to promptly identify the folding phase associated with C-SH3 (22). Notably, a semilogarithmic plot (chevron plot) of such a phase is perfectly superposable with C-SH3 measured in isolation (Fig. 1), which would already represent a signature of the robustness of the reaction.

To further depict the atomistic details of folding of C-SH3 in the SH2–SH3 construct, we report the chevron plots obtained for each of the produced variants in Figure 1, which represents a comprehensive side-by-side evaluation of the chevron plots obtained for each mutant analyzed independently and within the framework of its supramodular architecture. The corresponding folding and unfolding parameters can be found in Table 1. Notably, a striking consistency emerges across virtually all instances, with the chevron plots of these variants exhibiting remarkable similarity at both experimental conditions.

**Table 1:** Kinetic folding parameters of Grb2 SH2–SH3 WT and its site-directed variants

SH2–SH3	k_f_ (s^−1^)	m_f_ (kcal mol^−1^ M^−1^)	k_u_ (s^−1^)	m_u_ (kcal mol^−1^ M^−1^)	Φ
WT	10.2 ± 0.5	0.48 ± 0.02	0.12 ± 0.04	0.13 ± 0.03	
T1S	7.2 ± 0.4	0.48 ± 0.02	0.13 ± 0.03	0.16 ± 0.06	0.8 ± 0.7
Y2A	5.2 ± 0.7	0.9 ± 0.1	0.62 ± 0.06	0.09 ± 0.01	0.29 ± 0.08
V3A	3.5 ± 0.2	0.58 ± 0.04	0.24 ± 0.03	0.10 ± 0.01	0.6 ± 0.1
A5G	2.2 ± 0.5	0.9 ± 0.2	0.54 ± 0.04	0.07 ± 0.01	0.5 ± 0.1
L6A	6.9 ± 0.7	0.77 ± 0.07	1.29 ± 0.04	0.08 ± 0.01	0.14 ± 0.04
F7A	5.3 ± 0.3	0.55 ± 0.04	0.23 ± 0.03	0.10 ± 0.01	0.5 ± 0.1
F19A	1.5 ± 0.3	1.2 ± 0.1	1.44 ± 0.04	0.09 ± 0.01	0.43 ± 0.06
F24A	4.6 ± 0.3	0.73 ± 0.06	0.95 ± 0.06	0.06 ± 0.01	0.42 ± 0.07
I25V	4.6 ± 0.3	0.73 ± 0.06	0.95 ± 0.06	0.06 ± 0.01	0.28 ± 0.04
H26A	25 ± 1	0.54 ± 0.02	0.22 ± 0.07	0.13 ± 0.03	a
S31A	7.2 ± 0.3	0.46 ± 0.02	0.26 ± 0.06	0.14 ± 0.02	0.3 ± 0.1
A39G	15 ± 1	0.70 ± 0.05	1.2 ± 0.1	0.05 ± 0.01	−0.21 ± 0.06
H41A	10.5 ± 0.8	0.63 ± 0.04	0.8 ± 0.1	0.06 ± 0.01	−0.01 ± 0.05
T44S	9.3 ± 0.5	0.69 ± 0.04	0.75 ± 0.07	0.03 ± 0.01	0.05 ± 0.04
Y51A	12.0 ± 0.5	0.56 ± 0.02	0.46 ± 0.06	0.10 ± 0.01	−0.13 ± 0.06
T53S	9.5 ± 0.5	0.52 ± 0.02	0.28 ± 0.04	0.13 ± 0.01	0.08 ± 0.08
V55A	10.0 ± 0.4	0.56 ± 0.02	0.38 ± 0.04	0.09 ± 0.01	0.01 ± 0.05

a This mutant showed a ΔΔG_D-N_ <0.4 kcal mol^−1^, preventing a reliable calculation of the Φ value.

**Figure 2:**
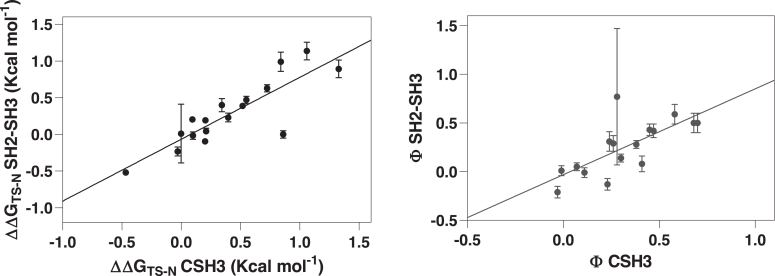
**ΔΔG plots and Φ–Φ plots for C-SH3 and SH2–SH3 tandem.** Each point in the graphs represents a single site-directed mutation occurring in both proteins. A strong linear correlation is evident for the analysis of both the Φ values and the ΔΔG_TS-N_.

One effective method for comparing mutational datasets involves creating Φ–Φ plots for a relevant state and examining the alterations in free energy resulting from mutations. In Figure 2, we present Φ–Φ and ΔΔG plots for the folding transition state of C-SH3 when analyzed separately and within the SH2–SH3 construct. Notably, the data for the transition state remain highly consistent between the two constructs, indicating a linear correlation with a slope of 1. On the basis of these observations, we conclude that the folding pathway of C-SH3 is highly robust, and it is not perturbed by the presence of its contiguous SH2 domain.

### The binding properties of SH3–SH2 tandem with Gab2 WT

To elucidate the binding mechanism of Grb2 SH3–SH2 tandem and Gab2, we resorted to conduct kinetic binding experiments using a stopped-flow apparatus, by rapidly mixing a fixed concentration of SH2–SH3 (2 μM) with increasing concentrations (ranging from 2 to 14 μM) of a peptide mimicking Gab2 from residues 503 to 524 (Gab2_503–524_). All the kinetic traces were fitted with a single-exponential equation to calculate the observed rate constants (*k*_obs_) that were subsequently plotted as a function of the concentrations of Gab2_503–524_. Data were analyzed using a linear equation (see Equation 2 in the Experimental procedures section), where the slope and *y*-axis intercept correspond to the microscopic association (*k*_on_) and dissociation rate constants (*k*_off_), respectively. The affinity was calculated as *K*_*D*_ = *k*_off_/*k*_on_.

**Figure 3:**
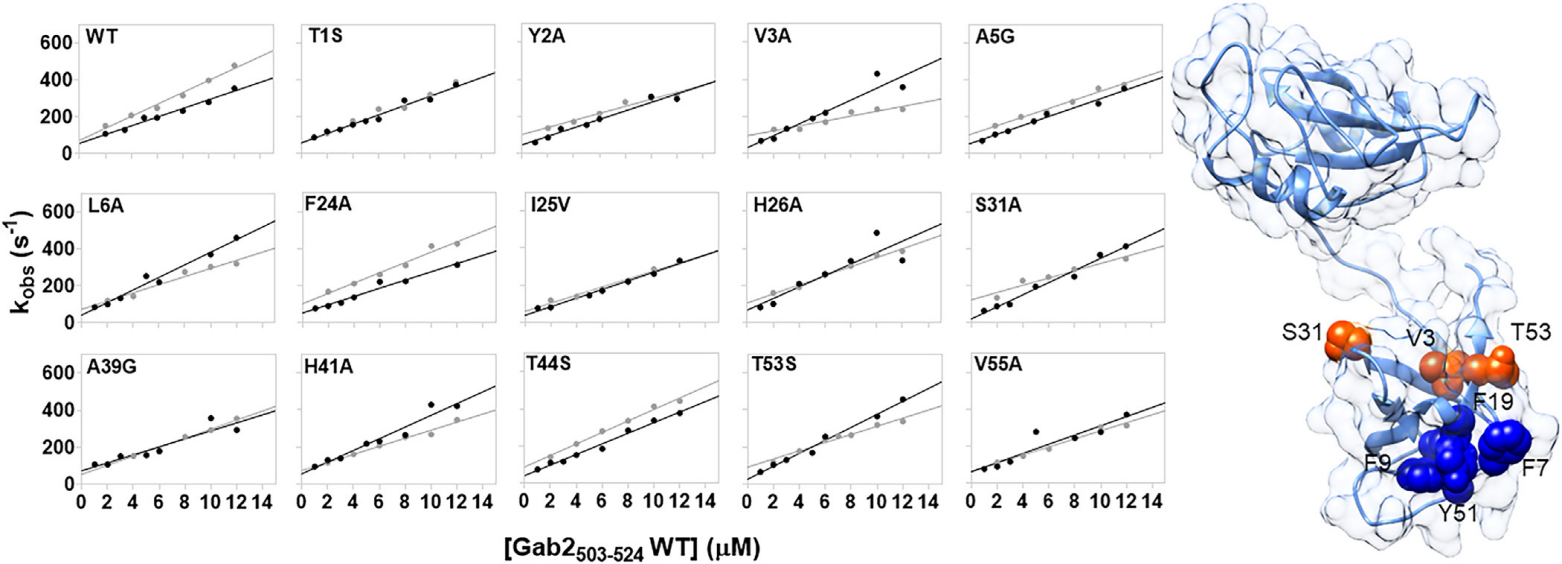
**Dependences of *k***_**obs**_**values of the binding reaction between site-directed mutants of SH2–SH3 (*gray*) and isolated C-SH3 domain (*black*) (data taken from Ref.** (12)**) at different concentrations of Gab2**_**503–524**_**.***Lines* represent the best fit to Equation 2. On the *right*, the *cartoon* representation of the tandem SH2–SH3 (Protein Data Bank code: 1GRI). The residues that displayed a very different change in affinity when compared with isolated C-SH3 domain are highlighted in *orange spheres* (see Table 2 and (12)). Interestingly, these positions are physically far from the binding pocket. Mutations that abrogate binding are highlighted in *blue spheres*.

As reported in Figure 3 and Table 2, the comparison between the kinetic binding experiment employing the WT-isolated C-SH3 domain (taken from Ref. (12)) and the WT SH2–SH3 tandem *versus* Gab2_503–524_ revealed minimal differences in terms of kinetic parameters, with a very similar calculated affinity for the peptide (*K*_*D*_ = 1.7 ± 0.1 μM for C-SH3 and *K*_*D*_ = 2.3 ± 0.5 μM for the SH2–SH3 tandem). This evidence would imply that the effect of the presence of the SH2 domain on the interaction between the C-SH3 domain and Gab2 is negligible. Nevertheless, to further investigate this aspect, we designed and reproduced the same site-directed mutations that we used in the study mentioned in Ref. (12) on the tandem SH2–SH3 construct. Then we measured the effect of mutations on the binding kinetics with Gab2_503–524,_ and we compared kinetic data with the ones previously obtained on the isolated C-SH3 domain. Intriguingly, mutations V3A, S31A, and T53S (highlighted in *orange spheres* in Fig. 3) resulted in a very different effect on *K*_D_ when measured on the tandem as compared with the isolated C-SH3 domain (Table 2 and (12)), implying a direct involvement of these positions in the binding of Gab2_503–524_. Curiously, however, these three residues are located at the interface with the SH2 domain and far from the SH3 binding pocket, suggesting the presence of a subtle interaction between the two domains during the binding reaction with Gab2_503–524,_ which demanded a further experimental investigation.

**Table 2:** Kinetic parameters of the binding reaction of Grb2 SH2–SH3 WT and its site-directed mutants with Gab2_503–524_ WT, Gab2_503–524_ P510A, and Gab2_503–524_ P512A

SH2–SH3	Gab2_503–524_ WT			Gab2_503–524_ P510A			Gab2_503–524_ P512A		
	*k*_on_ (μM^−1^ s^−1^)	*k*_off_ (s^−1^)	*K*_*D*_ (μM)	*k*_on_ (μM^−1^ s^−1^)	*k*_off_ (s^−1^)	*K*_*D*_ (μM)	*k*_on_ (μM^−1^ s^−1^)	*k*_off_ (s^−1^)	*K*_*D*_ (μM)
WT	32 ± 2	70 ± 16	2.3 ± 0.5	15 ± 1	95 ± 9	6.5 ± 0.8	13 ± 2	90 ± 17	7 ± 2
T1S	26 ± 2	65 ± 17	2.5 ± 0.7	20.3 ± 0.7	45 ± 5	2.2 ± 0.3	18 ± 2	86 ± 16	5 ± 1
Y2A	19 ± 2	102 ± 13	5.3 ± 0.8	22 ± 2	116 ± 16	5.1 ± 0.9	20 ± 2	87 ± 17	4 ± 1
V3A	13 ± 2	95 ± 17	7 ± 2	12 ± 2	99 ± 14	8 ± 2	11 ± 2	108 ± 21	9 ± 3
A5G	23 ± 2	104 ± 12	4.5 ± 0.6	15 ± 2	96 ± 16	6 ± 1	18 ± 3	125 ± 25	7 ± 2
L6A	22 ± 3	75 ± 21	3 ± 1	16.5 ± 0.3	86 ± 2	5.2 ± 0.2	19 ± 2	97 ± 18	5 ± 1
F7A^a^	—	—	—	—	—	—	—	—	—
F9A^a^	—	—	—	—	—	—	—	—	—
F19A^a^	—	—	—	—	—	—	—	—	—
F24A	28 ± 2	102 ± 19	3.7 ± 0.7	18 ± 1	127 ± 10	7.1 ± 0.7	18 ± 2	127 ± 12	7 ± 1
I25V	22 ± 2	59 ± 12	2.7 ± 0.6	27 ± 1	59 ± 8	2.2 ± 0.3	17 ± 2	62 ± 18	4 ± 1
H26A	24 ± 1	107 ± 11	4.4 ± 0.5	27 ± 2	105 ± 14	3.9 ± 0.6	14 ± 2	88 ± 18	6 ± 2
S31A	19 ± 3	126 ± 22	6 ± 2	24 ± 2	94 ± 20	3.8 ± 0.9	19 ± 2	110 ± 16	6 ± 1
A39G	24 ± 1	54 ± 11	2.2 ± 0.5	16 ± 2	99 ± 18	6 ± 1	17 ± 2	103 ± 13	6.1 ± 0.9
H41A	22 ± 2	73 ± 13	3.4 ± 0.6	16 ± 1	74 ± 10	4.5 ± 0.7	20.4 ± 0.9	82 ± 8	4.0 ± 0.4
T44S	31 ± 2	88 ± 12	2.8 ± 0.4	32 ± 2	68 ± 15	2.1 ± 0.5	21 ± 1	92 ± 9	4.4 ± 0.5
Y51A^a^	—	—	—	—	—	—	—	—	—
T53S	22 ± 2	91 ± 14	4.1 ± 0.7	15 ± 2	104 ± 20	7 ± 2	20 ± 1	110 ± 8	5.6 ± 0.5
V55A	22 ± 2	67 ± 13	3.1 ± 0.7	19 ± 2	85 ± 14	4.4 ± 0.8	17 ± 2	72 ± 12	4.3 ± 0.8

a In the case of these mutations, we could not obtain any reliable binding trace.

### Double mutant cycle analysis

**Figure 4:**
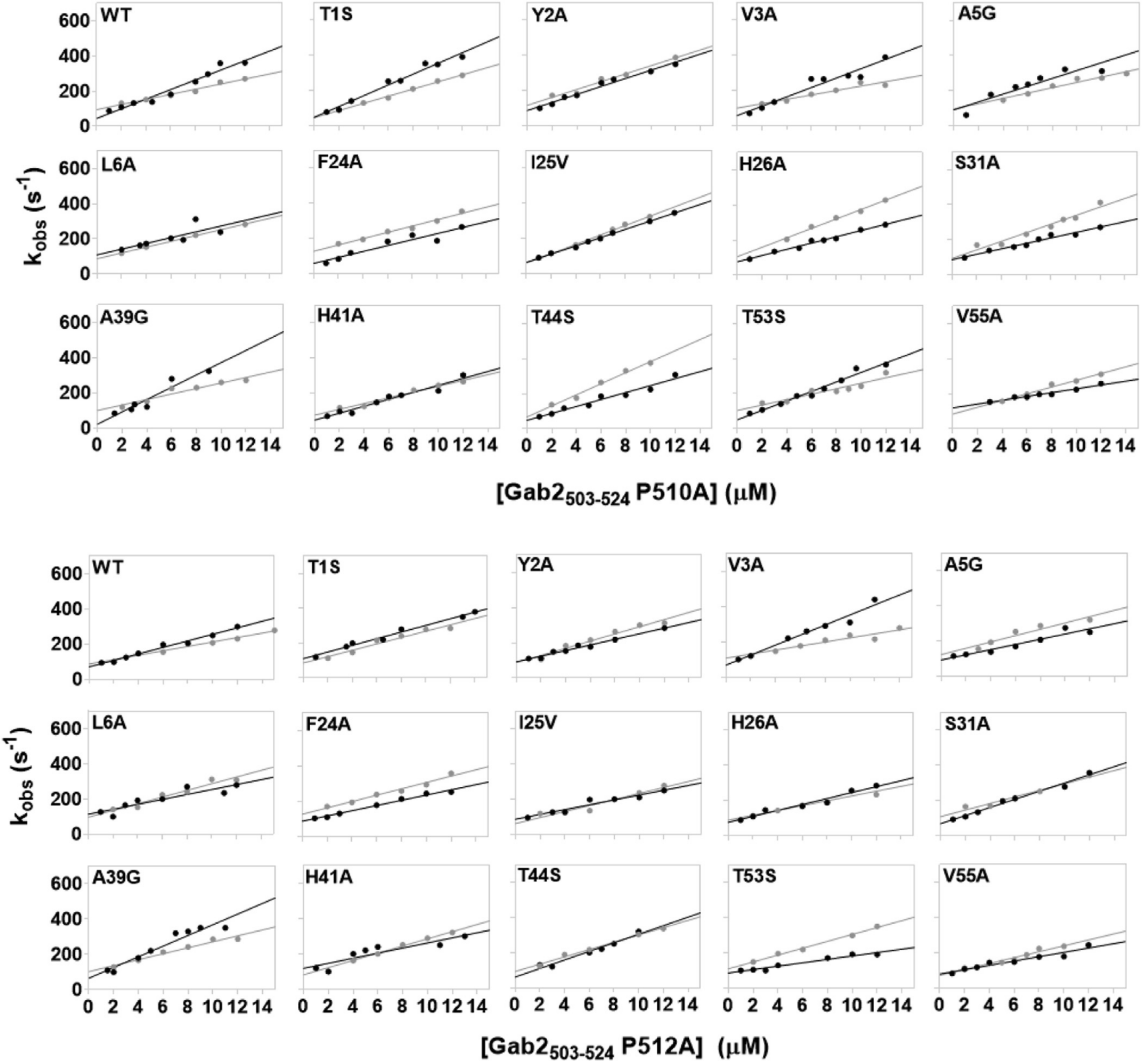
**Dependences of *k***_**obs**_**values of the binding reaction between site-directed mutants of SH2–SH3 (*gray*) and isolated C-SH3 domain (*black*) at different concentrations of Gab2**_**503–524**_**P510A (*top*) and Gab2**_**503–524**_**P512A (*bottom*).***Lines* represent the best fit to Equation 2.

To quantitatively characterize the role of residues located at the interface between the SH2 and the C-SH3 domain in the binding reaction with Gab2_503–524,_ we resorted to conduct a double mutant cycle analysis (the reader can find extensive dissertations about the rationale and methodology of this approach in Refs. (24, 25, 26)). In particular, we carried out kinetic binding experiments between all the tandem SH2–SH3 site-directed mutants *versus* different concentrations of two variants of the Gab2_503–524_ peptide, namely P510A and P512A. Plots of observed rate constants obtained at different concentrations of Gab2_503–524_ P510A and P512A are reported in Figure 4. We quantified coupling free energies ΔΔΔG by employing thermodynamic and kinetic parameters obtained from binding experiments involving the Grb2 tandem and both Gab2_503__–__524_ P510A and Gab2_503__–__524_ P512A peptides. The ΔΔΔG is calculated with the following equation:

$$\Delta \Delta \Delta G=\Delta \Delta G_{eq}^{Gab2WT}-\Delta \Delta G_{eq}^{Gab2mut}$$

**Figure 5:**
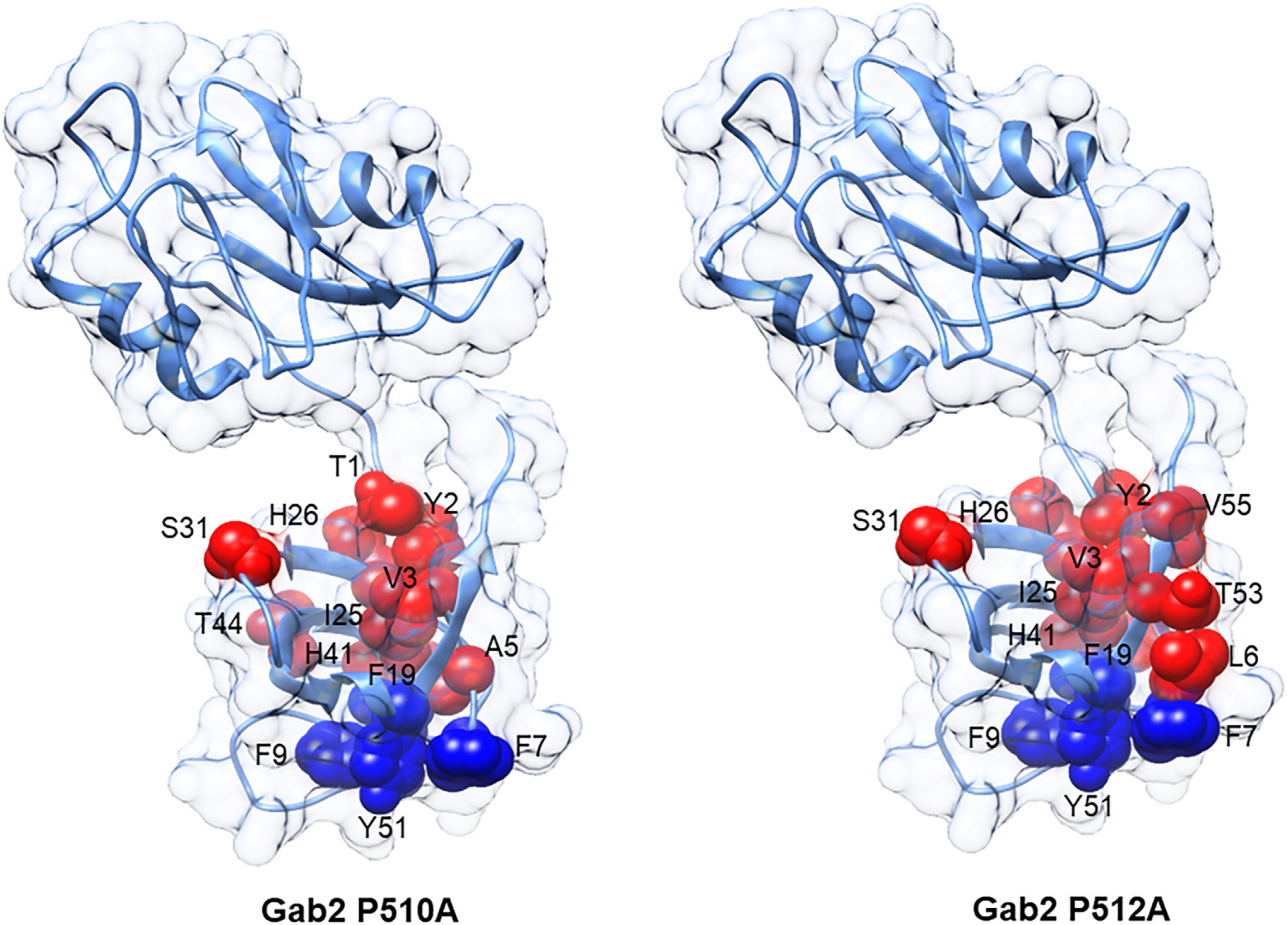
**Mutated residues (represented as *spheres*) of the tandem SH2–SH3.***Red spheres* indicate those residues reporting positive values of ΔΔΔG, whereas *blue spheres* highlight mutations that abrogate binding with Gab2_503–524_ P510A (*left panel*) and with Gab2_503–524_ P512A (*right panel*).

The calculated ΔΔΔG obtained for the tandem SH2–SH3 compared with those obtained for C-SH3 in isolation are reported in Table 3. Notably, we found that 9 of 14 residues (Thr1, Tyr2, Val3, Ala5, Ile25, His26, Ser31, His41, and Thr44) showed a detectable ΔΔΔG upon mutation and binding with Gab2 P510A, that is, with a value of ΔΔΔG ≥0.4 kcal mol^−1^. In particular, six of them, Tyr2, Val3, Ile25, His26, Ser31, and His41, were also found energetically coupled with the Gab2 P512A, with a ΔΔΔG of 0.8 ± 0.2, 0.5 ± 0.3, 0.5 ± 0.3, 0.4 ± 0.2, 0.7 ± 0.2, 0.5 ± 0.2 kcal mol^−1^, respectively. Residues showing a ΔΔΔG ≥0.4 kcal mol^−1^ are highlighted in *red* in Figure 5.

**Table 3:** Comparison of coupling free energies (ΔΔΔG) of the binding reaction of Grb2 SH2–SH3 mutants and Grb2 C-SH3 with Gab2_503–524_ P510A and Gab2_503–524_ P512A

Mutants	Gab2_503–524_ P510A		Gab2_503–524_ P512A	
	SH2–SH3	C-SH3	SH2–SH3	C-SH3
	ΔΔΔG (kcal mol^−1^)	ΔΔΔG (kcal mol^−1^)	ΔΔΔG (kcal mol^−1^)	ΔΔΔG (kcal mol^−1^)
T1S	0.7 ± 0.2	0.22 ± 0.08	0.3 ± 0.3	0.11 ± 0.06
Y2A	0.6 ± 0.2	−0.37 ± 0.07	0.8 ± 0.2	−0.13 ± 0.07
V3A	0.5 ± 0.2	−0.5 ± 0.1	0.5 ± 0.3	−0.1 ± 0.1
A5G	0.4 ± 0.2	0.09 ± 0.09	0.38 ± 0.25	−0.13 ± 0.07
L6A	0.35 ± 0.22	−1.1 ± 0.2	0.4 ± 0.3	−0.6 ± 0.2
F24A	0.2 ± 0.2	−0.28 ± 0.07	0.3 ± 0.2	−0.07 ± 0.06
I25V	0.7 ± 0.2	−0.3 ± 0.1	0.5 ± 0.3	−0.3± 0.1
H26A	0.7 ± 0.2	−0.21 ± 0.07	0.4 ± 0.2	0.26 ± 0.07
S31A	0.9 ± 0.2	−1.2 ± 0.1	0.7 ± 0.2	−0.4 ± 0.1
A39G	0.0 ± 0.2	0.4 ± 0.1	0.1 ± 0.2	0.86 ± 0.09
H41A	0.4 ± 0.2	−0.17 ± 0.08	0.5 ± 0.2	−0.04 ± 0.07
T44S	0.7 ± 0.2	−0.32 ± 0.08	0.38 ± 0.21	0.04 ± 0.09
T53S	0.3 ± 0.2	−0.6 ± 0.1	0.4 ± 0.2	−1 ± 0.1
V55A	0.39 ± 0.21	−0.81 ± 0.07	0.4 ± 0.2	−0.29 ± 0.07

It is of interest to investigate the structural distribution of residues that exhibit a detectable ΔΔΔG or a significant impact on binding. Since none of the previous residues are directly situated within the binding pocket of the C-terminal SH3 domain, we can deduce that they have an allosteric function in recognizing Gab2_503–524_, forming a sparse network within the tandem that modulates binding.

As described by the analysis of the DMC on the isolated C-SH3 domain (12), if a core residue mutation in the protein results in a smaller perturbation effect on peptide binding in the mutant compared with the WT protein, it suggests that the WT sequence is optimized for binding to the WT peptide sequence. This situation is characterized by positive ΔΔΔG values, meaning that for all mutated positions, the domain demonstrates both a highly conserved and an optimized structure. This, in fact, indicates that fine-tuning selectivity can be accomplished through long-range interactions, which are influenced by residues physically distant from the binding pocket.

## Discussion

The densely populated cellular environment, as well as the complex cellular surroundings, necessitates precise interactions among its many components to prevent any potential detrimental reaction. Nevertheless, it is intriguing to observe that despite the vast array of putative functions, the cell predominantly utilizes a restricted set of discrete protein–protein interaction domains, like the SH3 family. This particular family, despite its straightforward structure and a conserved binding pocket, plays various crucial roles in controlling disparate cellular events. The necessity for selectivity in the distinct functions of the different SH3 domains within cellular signaling underscores their significance despite their seemingly simple characteristics. In this context, it might be of interest to investigate whether the observed specificity arises, at least in part, from long-range effects that might arise both from intradomain and from interdomain energetic communication.

The comparison between the folding properties of the isolated C-SH3 and Grb2 SH3–SH2 tandem suggests that both the stability and folding pathway of C-SH3 are essentially insensitive to the presence/absence of SH2, which would imply the tandem to behave structurally as the sum of its parts, similarly to the beads-on-a-string model previously suggested in the case of acyl carrier domains (27), nucleosomes (28) or repetitive DNA clamps (29). Furthermore, when the binding properties of Grb2 C-SH3 and Grb2 SH3–SH2 are studied with a peptide mimicking WT Gab2 (Fig. 3, *top left*), only a small change is observed. In fact, the two constructs display a relatively marginal variation, mostly associated with an increase of the association rate constant that is at the limit of experimental detection. Hence, to a shallow analysis, addiction of the SH2 domain has nearly no effect in the stability, folding, and function of the SH3 domain. But, *the devil lies in the details*. The mutational analysis of Grb2 SH3–SH2 in fact highlights that the residues located at the interface between the two domains display a more pronounced change in *K*_*D*_ upon mutation when compared with the isolated C-SH3. This finding implies that the SH2 has some effect on the binding capabilities of C-SH3, which demands a careful investigation.

A powerful method to infer the presence of allosteric networks within a protein domain lies in performing a double mutant cycle. This technique allows quantifying the energetic coupling between two probed amino acids by comparing the effects of the double mutant to the sum of the two single mutants. Importantly, when the two mutations are designed in the protein and in the respective ligand, as exemplified in this work, double mutant cycles represent an effective method to measure the selectivity of the protein domain for that particular residue in the peptide ligand (24, 30). Previous analysis of protein–protein interaction domains by double mutant cycles suggested that this type of proteins do contain sparse energetic networks that may be important to modulate their functions. Notably, these findings have been observed on different nonhomologous domains, such as PDZ, SH2, SH3, and MATH domains (12, 18, 31, 32, 33, 34), indicating that the presence of sparse energetic networks might be a general property of protein–protein interaction domains. Importantly, since the ΔΔΔG reports on the effect of a mutation in recognizing the WT *versus* the mutated peptide, it might be interpreted as a signature of the *selectivity* of the domain to recognize the WT sequence. The comparative analysis presented in this work demonstrates that the coupling free energies obtained for the isolated C-SH3 and Grb2 SH3–SH2 tandem significantly differ, indicating that the distribution of the energetic network within the SH3 domain, and, therefore, its selectivity is strongly affected by the contiguous SH2 domain. This finding is in line with what recently observed in the case of the third PDZ domain of PSD-95 (9) and reinforces the importance of validating the results obtained on isolated domains with studies on more complex structural architectures.

The experimental data reported previously show that all the mutations reported in this study correspond to a positive coupling energy when considered both in reference to proline 510 in alanine and to proline 512 in alanine of the Gab2 peptide. That is, mutation of any core residue in the SH3 will affect the binding of the peptide such that the effect of mutating the proline of the peptide will be smaller in the mutant as compared with the WT. This compelling observation implies that the SH3 scaffold as a whole appears to be evolved to enhance ligand binding through intradomain allosteric coupling. Consequently, the selectivity of the SH3 is not only exclusively dictated by the specific residues directly engaged in ligand binding but also by the reminder of its globular fold. Remarkably, previous characterization of the C-SH3 in isolation did not observe such a homogeneous distribution of positive coupling energies (12), indicating that the contiguous SH2 domain plays a critical role in sculpting such allosteric communication.

## Conclusions

We have shown that despite highly robust stability and folding properties of the C-SH3 from Grb2, its binding selectivity, as probed by the presence and distribution of long-range allosteric networks, is profoundly affected by the presence of a contiguous SH2 domain. Notably, these effects are highly elusive and could only emerge from the comparison of an extensive mutational analysis. Hence, the results presented in this work appear particularly significant in at least two ways. First, we exemplify how the changes in allosteric networks might be subtle and elusive, emphasizing the importance of complementing works with isolated domains with experiments with more complex constructs. Second, we successfully identify an allosteric patch in Grb2, which mainly comprises the residues highlighted in Figure 5 and is located at the interface between C-SH3 and the SH2, which might represent an interesting target for the design of inhibitors that switch the selectivity of the SH3 domain without necessarily compromising its overall binding capability.

## Experimental procedures

### Site-directed mutagenesis

Grb2-SH2–SH3 was inserted into a pET28b+ plasmid vector. Constructs containing site-directed variants of SH2–SH3 were generated by utilizing the gene encoding Grb2-SH2–SH3 WT as a template. Site-directed mutagenesis was performed using the QuikChange Lightning Site-Directed Mutagenesis kit from Agilent Technologies, following the manufacturer's instructions. All mutations were verified through DNA sequencing.

### Protein expression and purification

The Grb2-SH2–SH3 construct and all the site-directed variants with N-terminal His tag were expressed in *Escherichia coli* BL21 (DE3) cells. Following an overnight culture, 10 ml of BL21 cells were used to inoculate 1 l of LB media conditioned with appropriate antibiotic (30 μg/ml kanamycin for plasmids). Bacterial cultures were subsequently incubated at 37 °C with constant agitation (180 rpm); when an absorbance of 0.7 to 0.8 at 600 nm was reached, 1 mM IPTG was added. The cultures were then cooled to 18 °C for 48 h to induce protein expression, and cells were then collected by centrifugation. To purify His tag proteins, the bacterial pellets were resuspended in 50 mM Tris–HCl buffer, 0.3 M NaCl, pH 7.5, and 10 mM imidazole with the addition of antiprotease tablets (Complete EDTA-free; Roche) and lysed by sonication. Cellular debris were removed by centrifugation at 11,000 rpm for 45 min at 4 °C, and proteins and the soluble fractions from bacterial lysates were loaded onto a nickel-charged HisTrap Chelating HP (GE Healthcare) column equilibrated with 50 mM Tris–HCl, 0.3 M NaCl, pH 7.5, and 10 mM imidazole. The proteins were then eluted with a gradient from 10 mM to 1 M imidazole by using an AKTA-prime system. Fractions containing the proteins were collected, and the buffers were exchanged to 50 mM Tris–HCl, 0.3 M NaCl, pH 7.5, by using a HiTrap Desalting column (GE Healthcare). The purity of the proteins was analyzed through SDS-PAGE. Protein concentrations were estimated by measuring the absorbance of tryptophan residue at 280 nm and calculated through the Beer–Lambert equation.

### Stopped-flow folding experiments

Experiments on the kinetics of unfolding and refolding were conducted using a single-mixing SX-18 stopped-flow instrument from Applied Photophysics. The fluorescence emission changes were monitored during the experiments, which were conducted at 25 °C in a 50 mM sodium phosphate buffer at pH 7.2, with urea employed as the denaturant.

The experiment utilized a 280 nm excitation wavelength, and the fluorescence emission light was recorded with a 320 nm cutoff glass filter. Typically, five individual traces were averaged for each denaturant concentration. The final concentration for Grb2 SH2–SH3 and its variants was typically 1 μM. Grb2 SH2–SH3 kinetic folding data were fitted using the following equation:

(1) $$k_{\text{obs}}=k_{F}\;\exp\left(-m_{F}\;\left[\text{urea}\right]/\text{RT}\right)+k_{U}\;\exp\left(-m_{U}\left[\text{urea}\right]/\text{RT}\right)$$

where *k*_obs_ is the observed rate constant, *k*_F_ and *k*_U_ are the folding and unfolding rate constants in the absence of denaturant, and m_F_ and m_U_ correspond to their associated m values.

### Stopped-flow binding experiments

SH2–SH3 Grb2 WT and its site-directed mutants were produced as previously reported. Kinetic experiments of binding were performed on a single-mixing SX-18 stopped-flow instrument from Applied Photophysics, recording the change of fluorescence emission. The excitation wavelength used was 280 nm, whereas the fluorescence emission was collected using a 320 nm cutoff glass filter. The binding experiments were carried out at 10 °C in pseudo–first-order condition mixing a constant concentration of SH2–SH3 Grb2 in the WT and mutated forms (2 μM) *versus* increasing concentrations of Gab2_503–524_ WT and its mutants P510A and P512A (ranging from 2 to 14 μM). For all measurements, the buffer used was 50 mM Hepes, 0.5 M NaCl, pH 7.0. Dependences of *k*_obs_ as a function of the concentration of peptide were fitted with the following linear equation:

(2) $$k_{\text{obs}}=k_{\text{on}}\;\left[\text{Peptide}\right]+k_{\text{off}}$$

## Data availability

All data are contained within the article.

## Conflict of interest

The authors declare that they have no conflicts of interest with the contents of this article.
